# 

**Published:** 1949-10

**Authors:** 


### Local Medical Notes

We desire to offer our congratulations to Professor and Mrs. James Swain on the occasion of their Golden Wedding.

B.M.A. prizes for Medical Students have been awarded to Mr. H. J. Bland and Miss M. M. C. Shaw.

J. N. P. Davies has been awarded the Northern Persian Forces Silver Medal for 1947.

Councillor A. J. M. Wright has been elected Chairman of the Bristol Health Committee.

Mr. A. J. M. Wright has been appointed Semon Lecturer for 1949. Lecture at 5 p.m. on Thursday, November 3rd, in the lecture hall of the Royal Society of Medicine, "**Tonsillar Function**".

The Phillips Prize for 1950 will be awarded on an essay, the subject of which shall be "**Relationship of Traumatic Occlusion to Paradontal Disease**". Candidates must submit their essays to the Professor of Dental Surgery not later than 1st May, 1950.

APPOINTMENTS

Dr. W. Hobson has been appointed Professor of Social and Industrial Medicine in the University of Sheffield.

Dr. S. H. Kingston has been appointed Consultant Dermatologist to the United Britstol Hospitals.

*University of Bristol*

Dr. J. H. S. Heller.—Professor of Pharmacology.

C. W. Ottaway, Ph.D., M.R.C.V.S.—Professor of Veterinary Anatomy.

J. A. Laing, B.Sc., Ph.D., M.R.C.V.S.—Lecturer in Veterinary Science.

A. Carlyle, B.V.Sc., M.R.C.V.S.—Lecturer in Veterinary Physiology.

S. W. Hinds, M.D., B.Sc., M.R.C.P., D.T.M. and H.—Lecturer in Preventive Medicine.

SCHOLARSHIPS AND PRIZES

*Markham Skerritt Memorial Prize.*—Dr. J. A. Fraser Roberts.

*Bristol Royal Hospital Board's Gold Medal.*—A. H. Levy.

*Bristol Royal Hospital Board's Silver Medal.*—Suzanne K. R. Clarke.

*David Rayner Prize in Gynæcology.*—Juno M. Smith.

*Hewer Pathology Prize.*—G. Walter.

*Francis Dudley Memorial Prize.*—G. B. Bennett and N. G. Sanerkin.

*Ophthalmology Prize.*—D. J. Brain.

*Phillips Prize.*—J. Sanders.

*G. F. Fawn Prize.*—Mr. L. M. Lloyd.

*Western Dental Manufacturing Company's Prize.*—J. H. Marshall.

*Open Entrance Scholarships.*—Catherine A. Clark, Lilian D. France.

*Exhibitions.*—Izotte R. Coulton, M. Reylance.

DEGREES AND DIPLOMAS

**University of Cambridge.**—M.D.: O. C. Lloyd, P. D. Samman.

**Royal College of Physicians.**—F.R.C.P.: J. A. Fraser Roberts. M.R.C.P.: T. J. Rendle Short.

**Royal College of Surgeons.**—F.R.C.S.: D. A. Sarsfield, Barbara F. Smith. D.L.O.: A. R. Rowe.

**Conjoint Board.**—D.C.H.: T. J. Rendle Short, A. P. Radford. D.P.M.: W. A. Heaton-Ward. D.P.H.: F. H. Bodman. D.T.M. and H.: G. H. Wattley.

